# Iron-dependent cell death as executioner of cancer stem cells

**DOI:** 10.1186/s13046-018-0733-3

**Published:** 2018-04-10

**Authors:** Bin Zhao, Xin Li, Ye Wang, Peng Shang

**Affiliations:** 1 School of Life Science, Northwestern Polytechnical University, Xi’an, Shaanxi 710072 China; 2 Research & Development Institute in Shenzhen, Northwestern Polytechnical University, Shenzhen, 518057 China; 3 Key Laboratory for Space Bioscience and Biotechnology, Institute of Special Environment Biophysics, School of Life Science, Northwestern Polytechnical University, Xi’an, Shaanxi 710072 China

**Keywords:** Iron, Cell death, Executioner, Cancer stem cells

## Abstract

This commentary highlights the findings by Mai, et al. that ironomycin, derivatives of salinomycin, exhibited more potent and selective therapeutic activity against breast cancer stem cells by accumulating and sequestering iron in lysosome, followed by an iron-mediated lysosomal production of reactive oxygen species and an iron-dependent cell death. These unprecedented findings identified iron homeostasis and iron-mediated processes as potentially druggable in the context of cancer stem cells.

## Background

Cancer stem cells (CSCs) represent a subset of cells that exhibit self-renewal and multi-differentiation properties and tumorigenic capacities [1]^.^ Accumulating evidences were shown that CSCs play crucial roles in tumour metastasis, relapse and chemo/radio-resistance [2]. Therefore, CSCs are considered to be promising targets for an effective therapeutic strategy.

Salinomycin, a natural product of *Streptomyces albus* strain, is a broad spectrum antimicrobial agent with the activities against gram-positive bacteria, fungi and coccidiosis worldwide [3]. More recently, studies have shown that salinomycin acted as an effective natural compound through high throughput screening in cancer stem cells [4, 5]^.^ However, explicit mechanisms underlying the anti-cancer effects of salinomycin and its ability to eradicate CSCs are still unclear.

Iron, an essential functional component of most organisms, fulfils a variety of important biological processes such as DNA replication, protein synthesis and cell respiration, which are essential for cell growth and proliferation. However, iron also can generate reactive oxygen species (ROS) via the Fenton reaction in redox cycling, which may cause damage to the membrane lipid and DNA. Therefore, iron may have a dual role on cells, by both stimulating cell growth and causing cell death, particularly a new form of cell death named ferroptosis [6, 7].

**Fig. 1:**
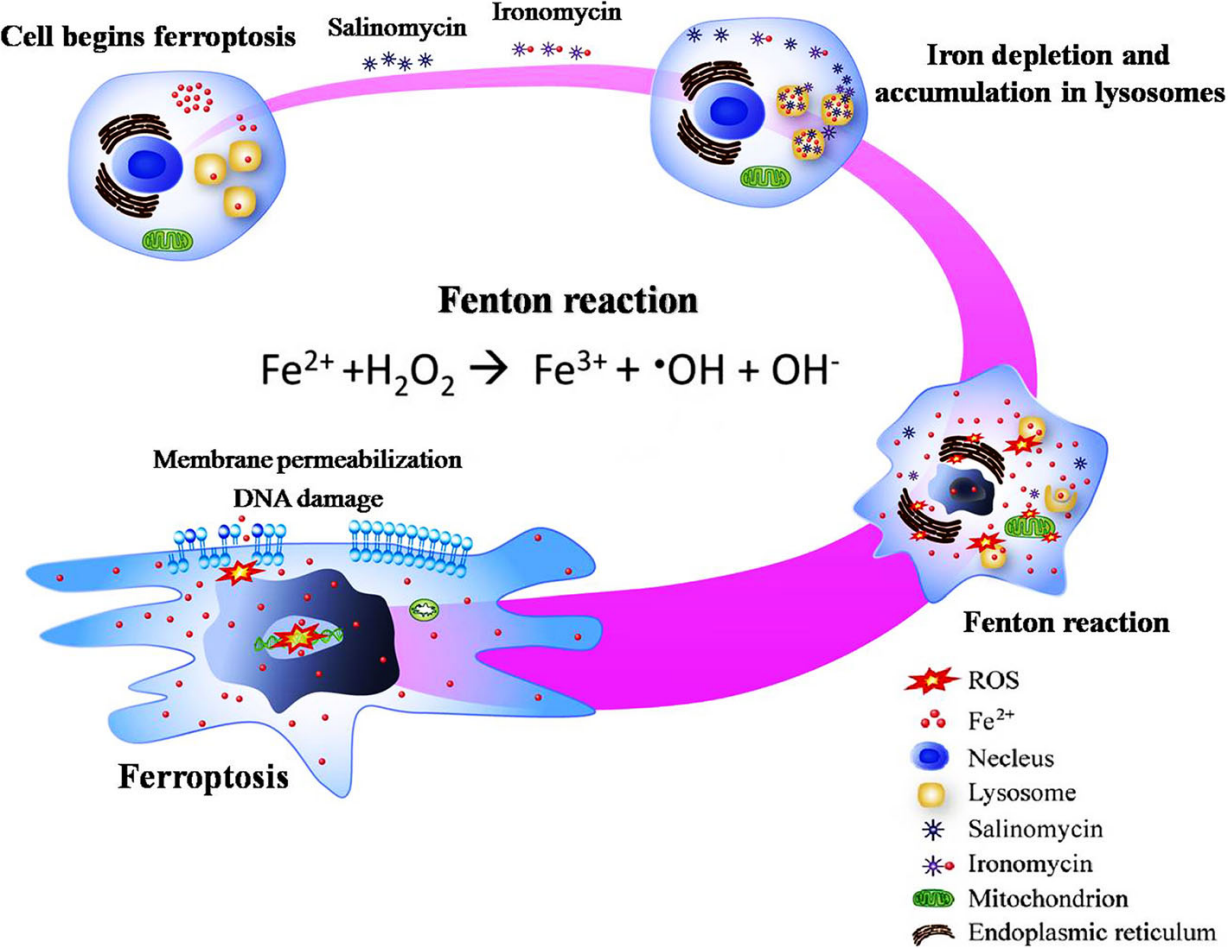
Schematic model of iron-dependent cell death. Salinomycin or ironomycin was physically accumulated by lysosomes of CSCs, as a result of iron overload followed by an iron-catalysed lysosomal production of ROS and lipid hydroperoxides via Fenton reaction, subsequent driven DNA damage and cell death that known as ferroptosis. Fenton reaction describes the redox reaction of iron (Fe^2+)^ with different peroxide species to generate hydroxyl (•OH) or alkoxyl (RO•) radicals. Ferric iron (Fe^3+^) can be recycled to Fe^2+^ by superoxide (•O_2_^−^) generating molecular oxygen (O_2_). The proposed mechanism was drawn by Bin Zhao and Xin Li based on Mai′ paper [8]

A recently published article by Mai, et al. [8] revealed that salinomycin, as well as ironomycin, operated as a selective agent against breast CSCs in vitro and in vivo. According to their investigation, they sought to generate a small library of structural variants with the original collections of salinomycin by a chemoselective oxidation. Further analyses indicated that ironomycin was physically accumulated in lysosomes, which induced iron loading in lysosomes and production of ROS via Fenton reaction. The implications of excess iron and ROS induced by ironomycin hinted towards the activation of ferroptosis (Fig 1). More importantly, the ironomycin displayed ten-fold higher potency against CSCs compared to salinomycin.

This study raised the question of the functional roles of iron in the therapy of cancer. In correlation with Mai′s results, two recent reports [9, 10] showed that USA Food and Drug Administration (FDA) approved iron nanoparticles can kill cancer cells through ferroptosis and apoptosis, offering new strategies of an iron age for cancer therapy. We appreciated their progressive work on anti-cancer therapies by regulating iron homeostasis; However, some issues should be considered. First, whether the ironomycin has the toxic effects on liver and kidney or other organs, since it is probably metabolized by liver and eliminated by kidney; Second, as discussed in the article, it is conceivable that iron is directly involved in the production of ROS, whether the excess iron-mediated ROS has certain damage to normal cells; Third, the specific mechanisms of salinomycin or ironomycin selectively killing cancer stem cells need to be clarified. Last but not least, even if they developed a drug that target cancer stem cells, that will not eliminate cancer. Of note, it would be worthwhile to develop extensive preclinical and clinical research.

## Conclusion

Taken together, these data demonstrated that iron and its derivates had therapeutic potential to hand over cancer stem cells through ferroptosis, however, there are no ongoing clinical trials. Further clinical outcomes are expected.

## Abbreviations

CSCs: Cancer stem cells
FDA: Food and Drug Administration
ROS: Reactive oxygen species
